# Presentations and Incidence of Ocular Injuries Caused by Motorcycle Accidents in Iraq

**DOI:** 10.12688/f1000research.142871.1

**Published:** 2024-03-11

**Authors:** Zainab A. Hashim, Suzan K. Mohammed‎, Marwan Y. Abdulla, Hayder A Fawzi

**Affiliations:** 1 College of ‎medicine, University of Al-Qadisiyah, Diwaniyah, Al-Qādisiyyah Governorate, Iraq; 2 College of ‎medicine‎, Al Iraqia University, Baghdad, Baghdad Governorate, Iraq; 3 Opthalmology, Al-Noaman ‎teaching hospital, Baghdad, Iraq; 4 Department of Pharmacy, Al-Mustafa ‎University ‎College, Baghdad, Iraq

**Keywords:** Motorcycles, Accidents, Eye Injuries, Visual Acuity

## Abstract

**Background:**

Motorcycle accidents can be particularly hazardous, as riders are exposed to various risk factors, such as high speeds, lack of protective enclosures, and limited safety features.

**Aim of the study:**

To describe the ocular injuries associated with motorcycled accidents presented to a tertiary center in Iraq.

**Patients and methods:**

A multicenter cross-sectional (survey) study that involved 335 cases of motorcycle accidents that presented with unilateral or bilateral ocular trauma. The study was carried out at Ibn Al-Haitham Teaching Hospital, Al-Nauman Teaching Hospital, Al-Diwaniyah Teaching Hospital, and forensic centers located in Baghdad (Iraq capital) and Al-Qadisiyah from 1 ^st^ of June 2019 to 1 ^st^ of June 2023. Information regarding ocular injuries was recorded and classified according to the International Ocular Trauma Classification.

**Results:**

The study involved 335 ophthalmological accidents; the mean age of the patients was 27.84± 9.6 years, most of them were males (96.7%), and there were only 11 females as passengers, 39 (11.6%) had injuries in both eyes. Lesions involving the periorbita, lids, and conjunctiva comprised most of the findings. There were 60.9% of patients with lid lacerations with or without sub-conjunctival bleeding, 22.1% with corneoscleral injury, and 17.9% with commotio retinae. It was the leading cause of decreased visual acuity, with 9% having lens capsule damage with or without iris prolapse and 8.1% having a ruptured capsule, 55.82% of patients had a mild injury, 27.16% had a moderate injury, and 17.01% had a severe injury.

**Conclusion:**

Eye injury associated with motorcycle accidents mainly affect males in their youth age and has serious outcomes, sometimes ending with blindness.

## Introduction

Millions of injuries occur worldwide as a result of road traffic accidents, making them the primary cause of death in adolescents and adults across the world. ^1^ Motorcycle-related accidents, in particular, pose a significantly higher risk of injury than other types of road traffic accidents. ^2^ In the American Academy of Ophthalmology, there is an annual occurrence of 2.5 million eye injuries in the United States, with around 50,000 individuals experiencing either partial or complete loss of vision. ^1^^,^^3^

While a relatively small proportion of eye injuries contribute to these statistics through motorcycle accidents, it is important to recognize that eye injuries and vision loss can occur as a result of motorcycle crashes. ^4^ Therefore, it is strongly advised that both motorcyclists and their passengers consistently wear appropriate eye protection whenever they ride their bikes. ^5^ Recent data from the United States indicates a 10.5% rise in such incidents in 2021 compared to 2020. ^6^ The problem of motorcycle injuries and fatalities has a greater impact on individuals from lower and middle socioeconomic classes, primarily due to motorcycles being a common mode of transportation in developing countries and to a lesser extent in developed nations. ^7^^,^^8^

The lack of strict enforcement of licensing regulations has created opportunities for individuals to obtain licenses without undergoing required eye tests or demonstrating riding proficiency. ^9^^,^^10^ Consequently, many motorcycle riders are unfamiliar with the rules and regulations outlined in the Highway Code in Iraq. ^11^ This has led to a high occurrence of accidents involving motor vehicles and motorcyclists, collisions between motorcyclists and pedestrians, and even among motorcyclists themselves. Intraocular injuries can be categorized into two types: closed globe injuries, which involve partial-thickness wounds in the wall of the eyeball ^12^ and can be further classified as contusions or lamellar lacerations, ^13^ and open globe injuries, which involve full-thickness wounds in the eyeball wall, such as ruptures caused by blunt trauma, ^12^ lacerations, penetrating or perforating injuries, or injuries involving intraocular foreign bodies resulting from sharp forces. ^14^ Adnexal injuries, on the other hand, pertain to injuries affecting the eyelid and/or conjunctiva. ^15^

Ocular trauma imposes a substantial burden on ophthalmic services, constituting 38-52% of all new patients seeking emergency care at hospitals due to accidents. ^15^^–^^17^ There is a limited amount of literature available regarding ocular injuries specifically related to motorcycle-related trauma. Enock *et al.* conducted a study involving 56 eyes with ocular injuries, and it was found that none of the patients were wearing helmets at the time of the injury. ^18^ Ocular trauma has the potential to cause significant and irreversible visual impairment due to the intricate and delicate structure of the eye. Ocular traumas are known to be the leading cause of monocular low vision and blindness. ^19^ The objective of the current study is to highlight the most common types of ocular injuries resulting from motorcycle accidents in Iraq, focusing on the severity of these injuries and their impact on visual outcomes.

## Methods

### Study design

In a multicenter cross-sectional (survey) study that involved 335 cases of motorcycle accidents that presented with unilateral or bilateral ocular trauma. Consent was obtained from the patients to participate in the study, and information on the age, sex, laterality, type of ocular injury, ocular findings, severity of the lesion, and visual acuity were recorded, these information regarding ocular injuries were recorded and classified according to the International Ocular Trauma Classification used in previous studies for the classification of ocular traumas according to visual acuity, pupillary light response, zone and type of injury, each given a score as illustrated by Dahal and Byenju (2013). ^20^

### Study settings

The study was carried out at Ibn Al-Haitham Teaching Hospital, Al-Nauman Teaching Hospital, Al-Diwaniyah Teaching Hospital, and forensic centers located in Baghdad (Iraq capital) and Al-Qadisiyah from 1 ^st^ of June 2019 to 1 ^st^ of June 2023. Written consent was obtained from the patients if they were fully oriented or from their caregivers (for participants aged below 18 since they are considered children in Iraq), after providing an adequate explanation of the aims and methods of the study.

### Participants

*Inclusion criteria*

Patients of all ages and both sexes are presented with ocular injuries during motorcycle accidents.

*Exclusion criteria*

Patients who refused to participate in the study.

### Ethical approval

This study was approved by the Research Ethics Committee in Al-Qadisiyah University/College of Medicine. (Approval no.: 25/285, on 22 ^nd^ of May 2019). Written consent was obtained from the patients if they were over 18 years old or from their caregivers, after providing adequate explanation of the aims and methods of the study.

### Sample size estimation

Sample size estimation was based on the following equation:

$$\text{minimum sample size}\;\left(n\right)=p\frac{\left(1-p\right)Z_{0.95}^{2}}{d^{2}}$$

Where n is the minimum sample size, p is the prevalence of ocular trauma was 13.5% based on a previous study ^21^ among patients admitted to the trauma unit. The Z represents the Z-score at 95 % confidence interval, and it equals 1.96, *d* represents the marginal error which is accepted according to Ref. 22 to be 0.04. Thus, the minimum sample size was estimated to be approximately 280.

### Statistical analysis

Descriptive statistics were used that included frequency and percentage; GraphPad Prism version 10 was used for the analysis.

## Results

The mean age of the patients was 27.84± 9.6 years, most of them were males (96.7%), and there were only 11 females as passengers, 39 (11.6%) had injuries in both eyes, as shown in Table 1.

**Table 1.** Characteristics of enrolled patients.

Characteristic	N (%)
Number of cases	335
Age	
<18 year	121 (36.1%)
18-50 years	142 (42.4%)
>50 years	72 (21.5%)
Sex	
Male	324 (96.7%)
Female *	11 (3.3%)
Eye involved	
One eye	296 (88.4%)
Both eyes	39 (11.6%)

* The involvement of females was as passengers.

Lesions involving the periorbita, lids, and conjunctiva comprised the majority of the findings. There were 60.9% of patients with lid laceration ± sub-conjunctival hemorrhage, 22.1% with corneoscleral injury, 17.9% with commotio retinae and it was the leading cause of decreased visual acuity, 9% with lens capsule damage ± iris prolapse, and 8.1% with ruptured capsule, as shown in Table 2.

**Table 2.** The categorization of patients according to type of presenting eye lesion.

Presenting lesion *	N (%)
Lid laceration ± subconjunctival hemorrhage	204 (60.9%)
Hyphema	90 (26.9%)
Cornea ± scleral wound	74 (22.1%)
Commotio retinae	60 (17.9%)
Lens capsule damage ± iris prolapse	30 (9.0%)
Rupture globe	27 (8.1%)
Diplopia (blunt trauma)	17 (5.1%)
Blowout fracture	13 (3.9%)

* This grouping concentrates on the presenting and most severe injury.

After applying the International Ocular Trauma Classification system, 55.82% of patients had a mild injury, 27.16% had a moderate injury and 17.01% had a severe injury, as shown in Table 3.

**Table 3.** Severity of lesion.

Severity	No (%)
Mild	187 (55.82%)
Moderate	91 (27.16%)
Severe	57 (17.01%)

The visual acuity of the participants ranged from 6/6 to no light perception, 59.1% of patients had visual acuity better than 6/18, 29.0% from 6/18 to 6/60, 8.1% from 6/60 to counting fingers, and 3.9% with no light perception, as shown in Table 4.

**Table 4.** Visual acuity assessment.

Snellen visual acuity	N (%)
Better than 6/18	198 (59.1%)
Between 6/18-6/60	97 (29.0%)
6/60-CF	27 (8.1%)
Non-seeing eye	13 (3.9%)

## Discussion

Ocular injuries resulting from motorcycle accidents can range from mild to severe and can have lasting effects on vision; motorcycle accidents often expose riders to various types of impact debris and forces that can directly or indirectly lead to ocular injuries. ^4^

The age and sex distribution in the current study were in agreement with the results of Larona and Pe-Yan (2012), as they studied 34 cases of motorcycle ocular injuries and reported a mean of 27.1 years, and 88.2% of their study sample was composed of males. ^23^ Hsieh *et al*. (2017) reported that 6,072 were admitted due to motorcycle injuries, 994 (16.4%) of them were ≥ 65 years, and 5078 (83.6%) were aged between 20-64 years; however, females formed 41.9% of the elderly and 42.9% of adults. ^24^ These findings offer a much higher rate of injury in females; the lower rate of females injured in the current study could be attributed to cultural hindrances posed by society, which limit females as the primary drivers of vehicles. Social/cultural factors play a significant role in the age and sex distribution of motorcycle riders, as in Taiwan it is very common for females to ride bikes, ^25^ and in another study done by the same author, female riders formed 42.01% of the study sample and were younger, passengers more than riders, used helmets more than males, and had different injury characteristics and lower in-hospital mortality in comparison to males. ^25^

In the current study, patient injuries included lid lacerations and sub-conjunctival hemorrhage, corneoscleral injury, commotio retinae, lens capsule damage ± iris prolapse, ruptured capsule, and orbital blowout fractures. Soft tissue injuries were the most prevalent probably because of the very limited use of helmets in our country. None of the current studies reported using helmets regularly, Arif *et al*. (2019) reported that although facial soft tissue injuries occur in helmeted riders, it occurs far more in non-helmeted ones. ^26^ To the best of our knowledge, no study directly reported open globe injury due to motorcycle injury, rather aggregated road traffic accidents cases, Upaphong *et al*. (2021) reported that motorcycle injuries constituted 56.3% of patients aged <20 years, 25% (age 20-39 years), 15% (aged 40-59 years), and 20% (age >60 years), and in all patients, hyphema were seen in 55.4%, lens injuries (52.5%), and uveal prolapse (55.4%). ^27^ In a review done by Kim *et al*. (2022) it was reported that blowout fractures were the third observed mechanism for orbital fractures, ^28^ Also other studies reported that motorcycle accidents were the leading cause of orbital/facial fractures. ^29^^,^^30^

Direct contact between the eye and a chemical ( *e.g.,* gasoline), or even chemical fumes, can cause burns. ^31^ Sources of radiation, such as bright ultra violet (UV) headlights, can also cause a flash burn. ^32^ Other drivers may be injured because of their reckless driving and collision with other cars and heavy objects on the side road. ^33^ Driving under the influence of alcohol can lead to accidents, which not only affects the driver but also the passengers and other people on the road. ^34^

### Limitations of the study

Since only two governates out of 18 limit the conclusion, at the same time, it gives us an initial assessment of the seriousness of the ocular injuries, a national study that involves most of the country is necessary.

## Conclusion

In summary, the analysis of eye injuries associated with motorcycle accidents highlights a significant concern primarily affecting young males. The outcomes of these incidents can be particularly severe, at times resulting in permanent blindness. Policymakers, traffic safety authorities, and healthcare professionals must address this issue with targeted interventions and awareness campaigns. By emphasizing the importance of proper protective gear, safe riding practices, and education, we can work towards reducing the occurrence of such injuries and minimizing their life-altering consequences. Furthermore, fostering a culture of responsible riding and enhancing road infrastructure can contribute to safer roads for all, ultimately preventing these devastating eye injuries and their far-reaching impacts on individuals and society as a whole.

## Data Availability

Zenodo: Ocular Injuries Caused by Motorcycle Accidents [Data set]. https://doi.org/10.5281/zenodo.8370156. ^35^ The project contains the following underlying data:
- Data sets of Ocular Injuries Caused by Motorcycle Accidents. Zenodo: Ocular Injuries Caused by Motorcycle Accidents. https://doi.org/10.5281/zenodo.8370208. ^36^ The project contains the following underlying data:
- STROBE Checklist. Data are available under the terms of the Creative Commons Attribution 4.0 International license (CC-BY 4.0).
