# Supplementary material for: Presentations and Incidence of Ocular Injuries Caused by Motorcycle Accidents in Iraq
Source: F1000Res. 2024 Aug 30;13:183. Originally published 2024 Mar 11. [Version 2] doi: 10.12688/f1000research.142871.2 (PMC11966094; doi:10.12688/f1000research.142871.2)
Supplement: Peer review history

# Reviewer report: Reviewer response for version 2

**DOI:** 10.5256/f1000research.170553.r373593

**Published:** 2025-04-02

**Authors:** Najah Mohammad

**Affiliations:** 1 University of Baghdad, Baghdad, Iraq

This article deals with the important subject of ocular injury by motorcycles; no previous study estimates the impact of these accidents in Iraq. Additionally, this study reports initial estimates regarding the type of injuries. Since it was carried out in multiple centers, this will provide a broader picture of the current problem in multiple centers.

 I have several minor questions:

 1/ Please calculate the 95% confidence interval of the overall prevalence of ocular injuries.

 2/ Why did you choose this particle type of equation to calculate the sample size, and what is the power of detection?

 3/ To better present your data, I recommend adding Figures, not just tables, as part of your results.

 4/ Please emphasize the study recommendations (Future perspective).

 Best regards

Is the work clearly and accurately presented and does it cite the current literature?

Yes

If applicable, is the statistical analysis and its interpretation appropriate?

Not applicable

Are all the source data underlying the results available to ensure full reproducibility?

Yes

Is the study design appropriate and is the work technically sound?

Yes

Are the conclusions drawn adequately supported by the results?

Yes

Are sufficient details of methods and analysis provided to allow replication by others?

Yes

Reviewer Expertise:

Ophthalmology

I confirm that I have read this submission and believe that I have an appropriate level of expertise to confirm that it is of an acceptable scientific standard, however I have significant reservations, as outlined above.

# Reviewer report: Reviewer response for version 2

**DOI:** 10.5256/f1000research.170553.r319026

**Published:** 2024-09-16

**Authors:** Debapriya Mukhopadhyay

**Affiliations:** 1 Debapriya Mukhopadhyay Vision Research Institute, Kolkata, India

The study is now a good read, Informative & comprehensive.

Is the work clearly and accurately presented and does it cite the current literature?

Yes

If applicable, is the statistical analysis and its interpretation appropriate?

Partly

Are all the source data underlying the results available to ensure full reproducibility?

Yes

Is the study design appropriate and is the work technically sound?

Yes

Are the conclusions drawn adequately supported by the results?

Yes

Are sufficient details of methods and analysis provided to allow replication by others?

Yes

Reviewer Expertise:

Public Health, Healthcare Strategy, Healthcare Administration, Community Eye Health, Diabetes, Medical imaging, Ophthalmology, Optometry, Medical Investigations & research, Cell biology research

I confirm that I have read this submission and believe that I have an appropriate level of expertise to confirm that it is of an acceptable scientific standard.

# Reviewer report: Reviewer response for version 1

**DOI:** 10.5256/f1000research.156471.r303232

**Published:** 2024-07-25

**Authors:** Debapriya Mukhopadhyay

**Affiliations:** 1 Debapriya Mukhopadhyay Vision Research Institute, Kolkata, India

What went well?

 The comprehensive study conducted across multiple centres in Iraq provides valuable insights into the ocular injuries resulting from motorcycle accidents. The meticulous classification of injuries according to the International Ocular Trauma Classification has allowed for a detailed understanding of the types and severities of ocular trauma. The findings highlight the importance of protective gear and safety measures for motorcyclists, as the majority of injuries were to young males, indicating a demographic at high risk. The study's extensive data collection over four years offers a solid foundation for developing strategies to prevent such injuries and improve outcomes for those affected. It's a significant step towards enhancing road safety and healthcare protocols for ocular injuries in Iraq and potentially other regions with similar contexts.

 The study gap

 The study presents a significant contribution to understanding the ocular trauma associated with motorcycle accidents in Iraq, highlighting the prevalence of such injuries among young male riders. However, the research reveals gaps that warrant further investigation. The disproportionate representation of male patients could be indicative of cultural or behavioral patterns that influence motorcycle usage and risk exposure, suggesting a need for gender-specific safety interventions. Additionally, the study's focus on tertiary centers in urban areas may not capture the full spectrum of ocular injuries, particularly those treated in rural or non-hospital settings. The classification of injuries, while standardized, does not account for long-term outcomes or the socioeconomic impact on the patients. Future studies could benefit from a longitudinal approach, assessing the recovery trajectory and quality of life post-injury, as well as exploring preventive measures tailored to the local context. Moreover, integrating multidisciplinary perspectives, including ophthalmology, trauma surgery, and public health, could provide a more comprehensive understanding of the factors contributing to these injuries and the strategies needed to mitigate them.

 Statistical significance is missing in the results.

Is the work clearly and accurately presented and does it cite the current literature?

Yes

If applicable, is the statistical analysis and its interpretation appropriate?

Partly

Are all the source data underlying the results available to ensure full reproducibility?

Yes

Is the study design appropriate and is the work technically sound?

Yes

Are the conclusions drawn adequately supported by the results?

Yes

Are sufficient details of methods and analysis provided to allow replication by others?

Yes

Reviewer Expertise:

Public Health, Healthcare Strategy, Healthcare Administration, Community Eye Health, Diabetes, Medical imaging, Ophthalmology, Optometry, Medical Investigations & research, Cell biology research

I confirm that I have read this submission and believe that I have an appropriate level of expertise to confirm that it is of an acceptable scientific standard, however I have significant reservations, as outlined above.

# Response

**Published:** 2024-08-17

**Authors:** Hayder A Fawzi

**Affiliations:** Pharmacy, Al-Mustafa University College, Baghdad, Iraq

Dear Reviewer,

**Reviewer Comments -**

 What went well?

 The comprehensive study conducted across multiple centres in Iraq provides valuable insights into the ocular injuries resulting from motorcycle accidents. The meticulous classification of injuries according to the International Ocular Trauma Classification has allowed for a detailed understanding of the types and severities of ocular trauma. The findings highlight the importance of protective gear and safety measures for motorcyclists, as the majority of injuries were to young males, indicating a demographic at high risk. The study's extensive data collection over four years offers a solid foundation for developing strategies to prevent such injuries and improve outcomes for those affected. It's a significant step towards enhancing road safety and healthcare protocols for ocular injuries in Iraq and potentially other regions with similar contexts.

 The study gap

 1/ The study presents a significant contribution to understanding the ocular trauma associated with motorcycle accidents in Iraq, highlighting the prevalence of such injuries among young male riders. However, the research reveals gaps that warrant further investigation. The disproportionate representation of male patients could be indicative of cultural or behavioral patterns that influence motorcycle usage and risk exposure, suggesting a need for gender-specific safety interventions.

***Author response***

 We agree with the reviewer the high prevalence of ocular injuries in males is attributed to couturier factors, such as males representing the majority of the drivers in our country, and which will lead to a higher proportion of males inflicted with ocular injuries.

**Reviewer Comments -**

 2/ Additionally, the study's focus on tertiary centers in urban areas may not capture the full spectrum of ocular injuries, particularly those treated in rural or non-hospital settings.

***Author response***

 We chose the largest hospitals in the country since other locations have low presentation of such access. We agree with the reviewer that there is a strong need to examine ocular injury in non-tertiary hospitals and other health care facilities. However, this is beyond the scope of the current study.

**Reviewer Comments -**

 3/ The classification of injuries, while standardized, does not account for long-term outcomes or the socioeconomic impact on the patients. Future studies could benefit from a longitudinal approach, assessing the recovery trajectory and quality of life post-injury, as well as exploring preventive measures tailored to the local context. Moreover, integrating multidisciplinary perspectives, including ophthalmology, trauma surgery, and public health, could provide a more comprehensive understanding of the factors contributing to these injuries and the strategies needed to mitigate them.

***Author response***

 We agree with the reviewer that this work should be the basis of further studies in this direction.

**Reviewer Comments -**

 4/ Statistical significance is missing in the results.

***Author response***

 This is a descriptive study; there is no comparison between the different groups, so no statistical analysis applies to the current work.

 Best regards

# Reviewer report: Reviewer response for version 1

**DOI:** 10.5256/f1000research.156471.r267248

**Published:** 2024-05-10

**Authors:** Japheths Ogendi

**Affiliations:** 1 Maseno University, Maseno, Kisumu County, Kenya

I have gone through the work. This is an important study that touches on a major public health concern, ocular injuries. However,  the study needs to be revised and additional information added to it to make it complete. For example, the background of the study needs to be revised to improve on its readability. I have provided details in track changes which can be viewed in the file linked here.

 The Section on Methods  needs to be reexamined. For example, the authors should make it clear to readers, who may not be familiar with the study settings, sufficient geographical to introduce  the study sites. Many readers may not be familiar with  Iraq or the teaching hospitals and forensic centers. Equally important is to add the reasons why the teaching hospitals and forensic centers were chosen. (I have included an example of a study that has shown how to do this in a reference indicated in track changes). It should also be made clear where exactly the study participants were gotten, were they Emergency Departments?  The authors should consider revising the findings and present them according to conventions of reporting scientific findings. The findings reported in this study started presenting ages and sex. It would be important for the readers to know the denominator, the total number of participants  then proceed to present the distribution different categories .

 The authors should strive to improve the discussion section by focusing the discussion on how the major findings of the study relates with other findings in previous studies. This has not been adequately achieved. The public health  implication of the study findings and the implication of the findings to practice and policy should be addressed where possible. Consider also revising the study limitations and draw three or four from study design, settings or data collection instruments.

 The work is a good effort but it can still be improved.

 I have also taken an effort to be more clear by adding proposed suggestions in track changes. If this is required Ill provide it. I think it has captured lots of details as well.

Is the work clearly and accurately presented and does it cite the current literature?

Yes

If applicable, is the statistical analysis and its interpretation appropriate?

Partly

Are all the source data underlying the results available to ensure full reproducibility?

Yes

Is the study design appropriate and is the work technically sound?

Partly

Are the conclusions drawn adequately supported by the results?

Yes

Are sufficient details of methods and analysis provided to allow replication by others?

Partly

Reviewer Expertise:

My areas of research are in Epidemiology with emphasis on Epidemiology of NCDs and road traffic injuries.

I confirm that I have read this submission and believe that I have an appropriate level of expertise to confirm that it is of an acceptable scientific standard, however I have significant reservations, as outlined above.

## References

[1] : Presentations and Incidence of Ocular Injuries Caused by Motorcycle Accidents in Iraq. *F1000Research* .2024;13: 10.12688/f1000research.142871.1 10.12688/f1000research.142871.1 PMC1196609440183006

# Response

**Published:** 2024-08-17

**Authors:** Hayder A Fawzi

**Affiliations:** Pharmacy, Al-Mustafa University College, Baghdad, Iraq

Dear Reviewer,

**Reviewer Comments -** 

 I have gone through the work. This is an important study that touches on a major public health concern, ocular injuries. However,  the study needs to be revised and additional information added to it to make it complete. For example, the background of the study needs to be revised to improve on its readability. I have provided details in track changes which can be viewed in the file linked

**Reviewer Notes from Tracked file -**

 1/ The reference needs to be counterchecked. The authors talk of the American Academy of Ophthalmology that has an annual occurrence of 2.5 million eye injuries. However, the references appear to be from other sources, not the academy. Kindly countercheck and update appropriately.

***Author response***

 Reference 3 was counterchecked, and it was inappropriately placed; below is the correct reference:

 American Academy of Ophthalmology and American Society of Ocular Trauma, United States Eye Injury Registry summary report, 1998–2002. Available at: http://www​.aao.org/newsroom​/guide/upload​/Eye-Injuries-BkgrnderLongVersFinal-l​.pdf

**Reviewer Notes from Tracked file -**

 2/ Also consider revising the sentence. Do the authors mean , for example, or could consider this kind of wordings: “ According to the America Academy of Ophthalmology, a total of 2.5 million eye injuries are reported/occur in USA annually, with 50,000 individuals experiencing either partial or complete loss of vision” (American Academy of Ophthalmology Report, 20..)

***Author response***

 We agree with the reviewer; the entire sentence lacks clarity; as such, we rephrase it to the following:

 According to the American Academy of Ophthalmology, 2.5 million eye injuries are reported/occur in the USA annually, with 50,000 individuals experiencing either partial or complete loss of vision.

**Reviewer Notes fromTracked file -**

 3/Regarding the number of previous studies on motorcycle accidents in Iraq,

***Author response***

 This is the first study to examine motorcycle ocular injury in Iraq; as such, no previous study can be reported. We used estimated facts from developing countries to reflect the numbers.

**Reviewer Comments -** 

 4/ The Section on Methods  needs to be reexamined. For example, the authors should make it clear to readers, who may not be familiar with the study settings, sufficient geographical to introduce  the study sites. Many readers may not be familiar with  Iraq or the teaching hospitals and forensic centers. Equally important is to add the reasons why the teaching hospitals and forensic centers were chosen. (I have included an example of a study that has shown how to do this in a reference indicated in track changes).

***Author response***

 We agree with the review suggestion; we included more details regarding the requested information as mentioned below:

 2.1 Study design

 This study presented motorcycle accident injuries patients to the Emergency Department from June 1, 2019, to June 1, 2023. After admission, patients were screened to identify the motorcycle injuries that presented ocular trauma; for those identified to have ocular trauma, written consent was sought and obtained from the patients to participate in the study. Information was obtained from the injured patients, whether they were stable enough or from those who accompanied them for care. Written consent was obtained from the patients if they were over 18 years old or from their caregivers after providing an adequate explanation of the aims and methods of the study. Further information was gathered on the age, sex, laterality, type of ocular injury, ocular findings, lesion severity, and visual acuity. The information regarding ocular injuries was classified and recorded according to the International Ocular Trauma Classification ^20^. Previous studies have used this classification and scoring method to classify ocular traumas according to visual acuity, pupillary light response, zone, and type of injury.

 2.2 Study settings

 According to the World Health Organization (WHO), Iraq's healthcare system has 2,652 facilities, of which 61 are teaching hospitals. This study was conducted in two governorates (Baghdad, the Iraqi capital, and Al-Qadisiyah), a total of three teaching hospitals: Ibn Al-Haitham Teaching Hospital (in Baghdad), Al-Nauman Teaching Hospital‎ (in Baghdad), and Al-Diwaniyah Teaching Hospital (in Al-Diwaniyah), and the three forensic centers associated with these hospitals. Patients were interviewed initially in the emergency department; if the ocular trauma related to motorcycle injuries were confirmed, the patient would be transferred to the ophthalmology department when the patient was managed and interviewed for the study.

 Ibn Al-Haitham Teaching Hospital is Iraq's primary and largest ophthalmology hospital. This hospital only receives patients with ophthalmological conditions. It has a large inpatient capacity (200 beds) and many operating rooms (16 Operating Rooms) for various ophthalmic procedures. Al-Nauman Teaching Hospital is one of the largest teaching hospitals in Baghdad, and it contains various departments, including emergency and ophthalmology departments. Finally, Al-Diwaniyah Teaching Hospital is the largest teaching hospital in the Al-Diwaniyah governorate, with large emergency and ophthalmology departments.

**Reviewer Comments -** 

 5/ It should also be made clear where exactly the study participants were gotten, were they Emergency Departments?  The authors should consider revising the findings and present them according to conventions of reporting scientific findings. The findings reported in this study started presenting ages and sex. It would be important for the readers to know the denominator, the total number of participants  then proceed to present the distribution different categories .

***Author response***

 The answer to this question is provided in the previous question.

**Reviewer Comments -** 

 6/ The authors should strive to improve the discussion section by focusing the discussion on how the major findings of the study relates with other findings in previous studies. This has not been adequately achieved. The public health  implication of the study findings and the implication of the findings to practice and policy should be addressed where possible. Consider also revising the study limitations and draw three or four from study design, settings or data collection instruments.

 The work is a good effort but it can still be improved.

***Author response***

 We improved both the results and the discussion accordingly.

 Abstract

 The study was conducted in three Teaching hospitals and forensic centers in Baghdad and Al-Qadisiyah in Iraq. Information regarding ocular injuries, recorded and classified according to the International Ocular Trauma Classification, was gathered from motorcycle crash injuries that were presented to the emergency departments of these facilities from June 1, 2019, to June 1, 2023.

 Results

 During the four years of the study, 6,243 patients presented with motorcycle injuries in the three Teaching hospitals and their forensic centers. Of those (5.37%), 335 patients were identified to have ocular trauma from the road crash. Table 1 presents the characteristics of motorcycle crash cases with ocular trauma. The ages of those with ocular trauma ranged from 10 to 67 years old, with a mean age of ‎27.84± 9.6‎ years. Most of them were 324 (96.7%) males, with a male-to-female ratio of 29 to 1, and there were only 11 females as passengers; 39 (11.6%) ‎had injuries in both eyes‎, as shown in Table 1.

 Regarding the type of eye lesions during their presentation, there were 60.9% of patients with lid laceration with or without sub-conjunctival hemorrhage, ‎‎22.1% with corneoscleral injury, 17.9% with commotio retinae, and it was the leading cause ‎of decreased visual acuity, 9% with lens capsule damage with or without iris prolapse, and 8.1% with ‎ruptured capsule, as shown in Table 2.‎

**Reviewer Comments -**

 7/ I have also taken an effort to be more clear by adding proposed suggestions in track changes. If this is required Ill provide it. I think it has captured lots of details as well.

***Author response***

 I appreciate your help; your notes and corrections improved the work, and we changed the article accordingly.

 Best regards
